# Supplementary material for: Identification of Novel Candidate Genes and Variants for Hearing Loss and Temporal Bone Anomalies
Source: Genes (Basel). 2021 Apr 13;12(4):566. doi: 10.3390/genes12040566 (PMC8069784; doi:10.3390/genes12040566)
Supplement: Supplementary file 1 [file genes-12-00566-s001.pdf]

TABLE S1. Rare damaging variants identified in 15 Filipino children with hearing loss requiring cochlear implants

Bold=probable cause of hearing loss; Italics=probable cause of other phenotypes

40.0%

ID	Age	Sex	AdditionalFeatures	FamilyHistory	Genotype	Variant	rsID	gnomAD_EAS(hg38)	gnomAD_highestMAF(hg38)	1000Genomes	GMEVariome	GenomeAsia100K_SEA	scaledCADD(hg38)	Damaging_dbNSFP41a	IMPC_gene/MouseHearingPhenotype	Literature on Known Phenotypes	Missed in previous analyses with hg19-refSeq/gnomAD or dbNSFP33a
1	3.95	M	EVA, L; brain cysts	.	**Heterozygous**	**DSPP:NM_014208:exon4:c.G730A:p.G244R**	**rs1044690454**	.	.	.	.	**0.001**	**24.3**	**FA/mLR/mSVM/MT/PP2/SI**	**NoEarPhenotype**	**PMID11175790: multiple Chinese families with hearing loss, one with EVA**	**No**
					Heterozygous	ANLN:NM_018685:exon11:c.G1921C:p.E641Q	rs748210737	0.0015	EAS:0.0015	.	.	.	20.9	MA/PP2	NoKOMouse	PMC6393648: heterozygous missense variant co-segregating with branchio-oto-renal syndrome in Chinese family	gnomAD MAF>0.001
					Potential Compound Heterozygous or Haplotype	ZNF462:NM_021224:[exon3:c.G3916A:p.G1306S];[exon3:c.G4129A:p.E1377K]	NA; rs768977077	NA; 0	NA; NFE:0.0001	.	.	.	28.9; 24.8	LRT/MT/PP2/SI; LRT/MA/MT/PP2/SI	Preweaning lethality	PMC6935050: hearing loss or inner ear malformation (not specified) in 50% of Weiss-Kruszka syndrome patients with heterozygous loss-of-function variants in exon 3 of ZNF462	No
					Heterozygous	*CEP290:NM_025114:exon39:c.C5284T:p.R1762C*	*rs373307908*	*0.0004*	*OTH:0.00096*	.	.	*0.013*	*29.6*	*LRT/MT/PP2/PR/SI*	*NoEarPhenotype*	*PMC2597962: heterozygous missense mutation in patient with Joubert syndrome including cystic kidney disease and hearing loss*	*No*
3	2.83	M	Malformed cochleae with incomplete cochlear turns, B; EVA, L; global developmental delay	.	**Heterozygous**	**LMX1A:NM_177398:exon5:c.G606C:p.L202F**	.	.	.	.	.	.	**24.8**	**FA/LRT/mLR/mSVM/MT/PP2/PR/SI**	**PMC3511360: KO-hom but not het mice have cochleovestibular defects**	**PMC5973959: heterozygous missense variants co-segregate with nonsyndromic hearing loss in two Dutch families; PMC6094940: homozygous missense variant co-segregates with nonsyndromic hearing loss in Pakistani family; PMID32840933: functional heterozygous missense variant in Korean proband**	**No**
					Potential Compound Heterozygous or Haplotype	USH2A:NM_206933:[exon47:c.G9286A:p.V3096M];[exon46:c.C9109T:p.R3037C]	rs147267500; rs770418427	0; 0.0002	AFR:0.0001; EAS:0.0002	.	.	0.007; 0.007	18.3; 23.8	MA/MT/SI/PP2; LRT/MA/MT/PR/SI	PMC1838616: Ush2a-KO mice have progressive photoreceptor degeneration and moderate nonprogressive hearing loss	MIM276901: AR Usher syndrome type 2A	Variant V3096M was ruled out in previous CLHNS screening due to MAF>0.01 (Truong et al. 2019)
					Heterozygous	***ZFHX4:NM_024721:exon10:c.G5119A:p.A1707T***	***rs764848741***	***0***	***FIN:0.0002***	.	.	.	***25.8***	***MT/PR/SI***	***NotPhenotyped***	***PMC3155189: 8q21.11 deletion including ZFHX4 in 8 patients with sensorineural hearing loss; developmental delay may be present***	***No***
					**Heterozygous**	**NRP1:NM_003873:exon10:c.G1655A:p.R552Q**	**rs757990959**	**0**	**OTH:0.0005**	.	.	.	**29.2**	**FA/LRT/MA/mLR/mSVM/MT/PP2/SI**	**Het-KO mice with abnormal ABR**	**PMC5695633: conditional-KO mice with disorganized outer spiral bundles and enlarged microvessels of stria vascularis, progressive hearing loss**	**No**
					**Heterozygous**	**COL2A1:NM_001844:exon50:c.G3569A:p.R1190H**	**rs748549541**	.	.	.	.	.	**27.3**	**FA/LRT/mLR/mSVM/MT/PP2/PR/SI**	**PMID10100048: Tg-mice with small misshapen otic capsule**	**MIM120140: multiple AD syndromes with hearing loss, including Stickler syndrome**	**No**
					Hemizygous	*ARHGAP4:NM_001164741:exon18:c.G2175C:p.Q725H*	*rs200637748*	*0.002*	*EAS:0.002*	*EAS:0.001*	.	.	*21.4*	*MT/PR/SI*	*NoEarPhenotype*	*PMID26707211: missense variant in child with intellectual disability; PMID22965914: deletion including ARHGAP4 in twins with intellectual disability*	*No; gnomAD MAF>0.001*
5	3.84	F	HJB with dehiscence, L; neonatal infection; pervasive developmental delay	.	*Potential Compound Heterozygous or Haplotype*	*CCDC186:NM_153249:[exon3:c.A461C:p.K154T];[exon3:c.G391A:p.A131T]*	*rs994643573; rs147799223*	*0.0004; 0.0004*	*EAS:0.0004; 0.0004*	.	.	*NA; NA*	*22.4; 15.4*	*LRT/MT/PP2/PR/SI; SI*	*Preweaning lethality*	*PMC7818090, PMC5502059: homozygous variants in patients with global developmental delay*	*Yes*
					**Heterozygous**	**DMXL2:NM_015263:exon3:c.T257C:p.L86S**	**rs761692429**	**0.0002**	**OTH:0.0005**			.	**24.1**	**LRT/MT/SI/PP2**	**Hom-KO Preweaning lethality, het-KO decreased bone mineral content**	**PMID27657680, PMID33715530: heterozygous missense variants cosegregating with AD hearing loss in Chinese and Cameroonian families**	**No**
					Heterozygous	ZFR2:NM_015174:exon19:c.C2653T:p.R885X	rs375337964	.	.	.	.	0.001	45	MT	NoKOMouse	PMID23610052: 19p13.3 deletions including ZRF2 in patients with intellectual disability or developmental delay, 40% with hearing loss	Yes
					Potential Compound Heterozygous or Haplotype	*MCM3AP:NM_003906:[exon25:c.G5383A:p.A1795T];[exon1:c.G998T:p.R333L]*	*rs17183290; rs17182552*	*0.004; 0.002*	*EAS:0.004; EAS:0.002*	*EAS:0.003; EAS:0.002*	.	*0.01; 0.01*	*26.7; 22.5*	*LRT/MA/MT/PP2/SI; MA*	*NoKOMouse*	*PMID32319184: AR Charcot-Marie-Tooth disease, including one Finnish patient with compound heterozygous variants and intellectual disability and hearing loss; PMC3477159: MCM3AP is required for inhibition of cellular DNA synthesis in viral infection*	*Yes; 1KG MAF>0.001*
6	10.81	M	PSCD + HJB, B; EVA, R; pneumonia, sinusitis, and progressive hearing loss	Hearing loss, paternal side	*Heterozygous*	*DNAH14:NM_001367479:exon58:c.G8842T:p.A2948S*	*rs541854543*	*0.0004*	*EAS:0.0004*	.	.	*0.003*	*23.9*	*PP2*	*NoKOMouse*	*PMID30125339: heterozygous deletion in patient with hydrocephalus and cognitive dysfunction; PMID33577779: compound heterozygous DNAH14 variants in Chinese proband with bronchiectasis, chronic sinusitis and otitis media*	*No*
					Heterozygous	ATAD2B:NM_017552:exon4:c.A475G:p.I159V	rs912934573	.	.	.	.	.	20.3	FA/mLR/MT	Hom-KO mice with abnormal ABR (het not tested)	.	No
					Heterozygous	ECT2:NM_001258315:exon15:c.G1579A:p.D527N	rs182472799	0.0002	SAS:0.001	EAS:0.001	.	0.01	21.2	LRT/MT	Het-KO mice with abnormal ABR	.	No; gnomAD MAF>0.001
					Heterozygous	TCOF1:NM_001135243:exon24:c.C3823T:p.R1275W	rs768512539	0	ASJ:0.0006	.	.	0 (SouthAsia=0.0007)	24.9	PP2/PR/SI	PMC489999: het-mutant mice with severe craniofacial defects, middle ear cavitation and hearing loss	MIM154500: AD Treacher-Collins syndrome often with conductive hearing loss and cleft palate	Previously ruled out due to lack of craniofacial features in patient carrying the variant (Truong et al. 2019)
					**Heterozygous**	**PTPRQ:NM_001145026:exon40:c.T6179C:p.V2060A**	**rs375150180**	**0.00097**	**EAS:0.00097**	**EAS:0.003**	.	**0.017**	**27.8**	**MT/SI**	**PMC6740823: rapid postnatal deterioration in cochlear hair-bundle structure**	**PMC5993672: AD nonsyndromic hearing loss**	**Yes**
					Heterozygous	*LRRK1:NM_024652:exon2:c.C16T:p.Q6X*	*rs772698984*	.	.	.	.	.	*33*	*MT*	*PMC4472125: Lrrk1-KO mice have severe osteopetrosis*	*PMC5348726: LRRK1 deficiency in humans and mice lead to increased bone mineral density, reduced body length and bone marrow, and sclerosis of vertebral endplates and pelvis; PMC5769692: reports of AR osteosclerotic metaphyseal dysplasia due to homozygous LRRK1 variants*	*No*
7	8	F	HJB, L; OM, L; mild motor delay and hypotonia; history of urinary and upper respiratory tract infections	Older brother seen for giftedness; Down syndrome, maternal cousin; asthma, paternal side	*Heterozygous*	*CPS1:NM_001875:exon27:c.3337-1G>T*	.	.	.	.	.	.	*34*	*MT*	*Preweaning lethality*	*MIM237300: AR carbamoyl phosphate synthetase I deficiency; PMID26440671: includes hypertonia at presentation but may also have hypotonia and mild motor delay later*	*No*
					Heterozygous	*GMPPB:NM_013334:exon8:c.G1032C:p.E344D*	.	.	.	.	.	.	*23.9*	*MT/SI*	*NotPhenotyped*	*PMC3710768,PMC4843780: two patients with compound heterozygous GMPPB variants had muscle weakness, intellectual disability and sensorineural hearing loss*	*No*
					Heterozygous	*TNXB:NM_019105:exon5:c.C2461T:p.R821X*	*rs749890642*	*0*	*NFE:0.00006*	.	.	*0 (Oceania=0.007)*	*36*	*LRT/MT*	*NoEarPhenotype*	*MIM600985: AR Ehlers-Danlos syndrome (EDS), AD vesicoureteral reflux; PMID27253074: conductive or sensorineural hearing loss common in EDS*	*No*
					Heterozygous	PLEC:NM_201380:exon20:c.2868+1G>A	rs368904034	.	.	.	.	0.001	34	MT	NotPhenotyped	MIM601282: AR/AD epidermolysis bullosa simplex, AR limb-girdle muscular dystrophy	No
					**Heterozygous**	**ARHGAP21:NM_020824:exon7:c.G475T:p.D159Y**	**rs1266442941**	**0.0002**	**EAS:0.0002**	.	.	.	**29**	**LRT/MT/PP2/PR/SI**	**Het-KO mice with abnormal ABR**	**PMC6406497: candidate gene for autism**	**No**
					**Potential Compound Heterozygous or Haplotype**	**[PCDH15:NM_001354411:exon29:c.C3787T:p.P1263S];[CDH23:NM_022124:exon27:c.G3262A:p.V1088M]**	**rs775954124; rs200632520**	**0.004; 0.002**	**EAS:0.004; EAS:0.002**	.	.	**NA; 0.003**	**24.9; 24.3**	**MA/MT/PP2/PR/SI; LRT/MA/mLR/mSVM/MT/PP2/SI**	**PMC2858222: double-heterozygous mice with progressive hearing loss**	**PMC2858222: compound heterozygous probands with congenital profound hearing loss**	**No; gnomAD MAF>0.001**

					**Heterozygous**	**MYO7A:NM_000260:exon36:c.G4921A:p.E1641K**	**rs767975012**	**0.0002**	**EAS:0.0002**	**.**	**.**	**0.003**	**26.2**	**LRT/MT/PP2/PR**	**AbsentPinnaReflex/AbnormalBone Mineralization(Hom); PMID15389316: mice heterozygous for missense variant have abnormal stereocilia bundles and low-frequency hearing loss**	**MIM276903: DFNA11; DFNB2; Usher syndrome IB**	**No**
					**Heterozygous**	**DNAJC17:NM_018163:exon11:c.A871G:p.I291V**	**.**	**.**	**.**	**.**	**.**	**.**	**18.96**	**MA/MT/SI**	**Het-KO mice with abnormal ABR**	**PMID26355662: homozygous variant in family with retinitis pigmentosa and hypogammaglobulinemia**	**No**
					Potential Compound Heterozygous or Haplotype	*NEO1:NM_002499:[exon3:c.G586C:p.D196H]; [exon24:c.C3443T:p.S1148F]*	*NA; rs1048863959*	*NA; 0*	*NA; LAT:0.00007*	*.*	*.*	*.*	*22.5; 25.9*	*MT; LRT/MT/PP2/PR/SI*	*NoKOMouse*	*PMID31953991: included in critical 15q24 microdeletion associated with autism and variable hearing loss, recurrent infections*	*No*
					Potential Compound Heterozygous or Haplotype	PIEZO2:NM_022068:[exon38:c.G5888A:p.R1963Q]; [exon37:c.C5557T:p.R1853C]; [exon9:c.A1159G:p.S387G]	rs1480686209; rs569343097; rs371514584	0; 0.0002; 0	AFR:0.00002; ASJ:0.0003; SAS:0.0002	NA; EUR:0.001; NA	.	NA; 0.001 (Oceania=0.03); 0.01 (Oceania=0.10)	25.6; 22.6; 24.6	MA/MT/PR/SI; SI; LRT	Preweaning lethality	PMC5097934: biallelic variants in probands with arthrogryposis, respiratory insufficiency, muscular atrophy, scoliosis and mild distal sensory loss	No
8	3.03	M	SSCD, L	.	**Heterozygous**	**COL11A1:NM_080629:exon58:c.A4364C:p.K1455T**	**rs769350133**	**0.0004**	**EAS:0.0004**	**.**	**.**	**.**	**28.6**	**FA/LRT/mLR/mSVM/MT/PP2/PR/SI**	**DecreasedStartleReflex**	**MIM120280: DFNA37; AD Marshall Syndrome; AD Stickler**	**No**
					Heterozygous	ZFHX4:NM_024721:exon3:c.G3007A:p.A1003T	rs369411377	0.002	EAS:0.002			0.009	27.8	MT	NotPhenotyped	PMC3155189: 8q21.11 deletion including ZFHX4 in 8 patients with sensorineural hearing loss	No; gnomAD MAF>0.001
					**Heterozygous**	**TECTA:NM_005422:exon10:c.C2967A:p.H989Q**	**rs200821009**	**0.003**	**EAS:0.003**	**EAS:0.003**	**.**	**0.001**	**20.4**	**FA/LRT/mLR/mSVM/MT/PP2/PR/SI**	**PMC2869304: mice heterozygous for missense mutation have non-functional outer hair cells**	**MIM602754: DFNA8/12; DFNB21**	**No; gnomAD MAF>0.001**
					Heterozygous	CEP290:NM_025114:exon48:c.G6629A:p.R2210H	rs371833544	0.0002	ME:0.003	.	.	.	23.2	LRT/MA/MT/PP2/PR/SI	NoEarPhenotype	PMC2597962: heterozygous missense mutation in patient with Joubert syndrome including cystic kidney disease and hearing loss	No
9	8.19	F	EVA, L	.	**Heterozygous**	**IST1:NM_001270976:exon8:c.C737G:p.P246R**	**rs774343604**	**0.0002**	**EAS:0.0002**	**.**	**.**	**.**	**24**	**LRT/MT/PP2/PR/SI**	**Het-KO mice with abnormal ABR**	**.**	**No**
13	5.95	M	Global developmental delay	Hearing loss, 3rd-degree cousin	Potential Compound Heterozygous or Haplotype	GDAP2:NM_017686:[exon14:c.A1463G:p.Y488C]; [exon2:c.C44T:p.T15I]	NA; rs1272520325	NA; 0	NA; AFR:0.00002	.	.	.	22.8; 19.8	MT/SI; LRT	AbsentPinnaReflex(Hom)	PMC7534050: AR adult-onset spinocerebellar ataxia	No
					**Heterozygous**	**SLC12A2:NM_001046:exon21:c.G2977T:p.E993X**	**.**	**.**	**.**	**.**	**.**	**.**	**60**	**MT**	**PMID10369265: Slc12a2-mice are deaf**	**MIM600840: DFNA78; AD Delpire-McNeill syndrome (global developmental delay and sensorineural hearing loss); AR Kilquist syndrome**	**No**
					Homozygous	ARMC4:NM_018076:exon5:c.A647G:p.N216S	rs981525890	0.0002	EAS:0.0002	.	.	0 (Oceania=0.03)	13.99	SI	NoEarPhenotype	PMC3788828: AR primary ciliary dyskinesia, may include hearing loss due to primary ciliary dyskinesia	No
18	2.77	M	.	.	Heterozygous	RERE:NM_012102:exon10:c.T910C:p.S304P	.	.	.	.	.	0.001	27.6	MT/PP2/SI	PMC3581587: heterozygous mice had decreased startle response and elevated ABR thresholds	PMC5903952: AD sensorineural hearing loss with eye, heart and renal defects	No
					Heterozygous	KRIT1:NM_194456:exon5:c.C74T:p.S25F	rs779446044	0	OTH:0.0005	.	.	0.003	28.6	FA/LRT/MA/mLR/mSVM/MT/PR/ PP2/SI	Het-/hom-KO have abnormal otic vesicle morphology	MIM604214: AD cavernous malformations	No
					Potential Compound Heterozygous or Haplotype	CDH23:NM_022124:[exon11:c.G982A:p.A328T]; [exon20:c.G2236A:p.V746I]	rs374545987; rs550384315	0.00096; 0.001	EAS:0.00096; EAS:0.001	AMR: 0.0014; AMR: 0.0014	.	0.02; 0.01 (Oceania=0.06)	28.4; 22.9	LRT/MT/PP2/SI; LRT?PP2/MT	Hom-KO mice with absent pinna reflex	MIM605516: DFNB12; AR Usher 1D	V746I was excluded due to CLHNS MAF=0.19 (Truong et al. 2019)
					**Heterozygous**	**MYO7A:NM_000260:exon36:c.G4921A:p.E1641K**	**rs767975012**	**0.0002**	**EAS:0.0002**	**.**	**.**	**0.003**	**26.2**	**LRT/MT/PP2/PR**	**AbsentPinnaReflex/AbnormalBone Mineralization(Hom); PMID15389316: mice heterozygous for missense variant have abnormal stereocilia bundles and low-frequency hearing loss**	**MIM276903: DFNA11; DFNB2; Usher syndrome IB**	**No**
					Potential Compound Heterozygous or Haplotype	NFRKB:NM_006165:[exon22:c.C3191G:p.T1064S]; [NM_006165:exon20:c.C2408T:p.P803L]	rs559022552; rs557840039	0.003; 0.003	EAS:0.003; EAS:0.003	EAS:0.002; EAS:0.002	.	.	19.7; 24.4	LRT; LRT/MT/PP2/SI	NoKOMouse	PMC2648978: NFRKB included in submicroscopic deletion in 11q24-25 in patients with hearing loss and multiple anomalies	No
19	5.66	F	Malformed cochleae, vestibules and semicircular canals, B; absent cochlear and inferior vestibular nerves, R	.	Heterozygous	PPOX:NM_000309:exon5:c.A394T:p.R132W	rs771307571	0.0002	EAS:0.0002	.	.	.	24.5	FA/mLR/mSVM/MT/PP2/PR/SI	Het-KO mice with abnormal ABR	MIM600923: AD porphyia variegata (1 case report of sudden hearing loss in a porphyria variegata patient)	No
					Potential Compound Heterozygous or Haplotype	GOLGB1:NM_001256486:[exon14:c.G7472A: p.R2491Q]; [exon14:c.A7367C:p.E2456A]; [exon14:c.C7112G:p.S2371C]	rs767375937; rs751019606; rs919070773	0; 0.0002; NA	AFR:0.00002; EAS:0.0002; NA	.	.	.	24.8; 25.9; 26.2	LRT/MA/MT/PP2/SI; LRT/MA/MT/PP2/PR/SI; LRT/MA/MT/PP2/SI	PMID2185186: osteochondrodysplasia; PMC4958322: palatal shelf defects	.	No
					Heterozygous	SLC36A1:NM_078483:exon7:c.C721T:p.Q241X	.	.	.	.	.	.	47	LRT/MT	KO-het abnormal craniofacial morphology	.	No
					Heterozygous	TFEB:NM_001167827:exon2:c.C136G:p.H46D	.	.	.	.	.	.	22.6	MT/PR/SI	Het-KO mice with abnormal ABR; decreased BMD	*PMCID: attenuates spiral ganglion neuron degeneration*	No
					Heterozygous	GDPD5:NM_030792:exon7:c.C404T:p.T135M	rs373413383	0	AFR:0.00002	.	.	0.01 (Oceania=0.07)	24.8	LRT/MA/MT/PP2	Hom-KO mice with abnormal ABR (het not tested)	.	No
					**Heterozygous**	**MYO18B:NM_032608:exon13:c.C2555T:p.A852V**	**.**	**.**	**.**	**.**	**.**	**.**	**26.1**	**FA/LRT/mLR/mSVM/MA/MT/PP2/PR/SI**	**Het-KO mice with abnormal ABR**	**MIM616549: AR Klippel-Feil syndrome with nemaline myopathy and facial dysmorphism; PMC5885878: hearing loss common in KFS; PMID18722888: 60% of Klippel-Feil HL patients with ear anomalies including inner ear dysplasia and deformed internal acoustic canal**	**No**
20	14.59	F	Fluctuating hearing loss with steeply sloping audiogram prior to CI; turbinate hypertrophy, allergic rhinitis, nasopharyngeal nodule	.	Heterozygous	ARHGAP29:NM_004815:exon17:c.G1847A:p.R616H	rs374991521	0.001	OTH:0.001	.	.	.	25.3	FA/MA/mLR/mSVM/MT/PP2/PR/SI	Het-KO mice with abnormal ABR	PMC3501616: cleft lip and palate	No; gnomAD MAF>0.001
					Heterozygous	CACNA1S:NM_000069:exon12:c.G1745C:p.G582A	rs377459546	0.0008	LAT:0.001	.	.	0.004	26	FA/LRT/MA/mLR/mSVM/MT/PP2/PR/SI	Het-KO mice with abnormal ABR	MIM170400: AD hypokalemic periodic paralysis	No; gnomAD MAF>0.001
					Heterozygous	DSPP:NM_014208:exon5:c.G1577A:p.S526N	rs201485801	0.001	EAS:0.001	EAS:0.003	.	0.02	13	FA/mLR/mSVM/PP2/SI	NoEarPhenotype	PMID11175790: multiple Chinese families with hearing loss, one with EVA	No; gnomAD MAF>0.001
					Heterozygous	CEP290:NM_025114:exon8:c.G503A:p.R168H	rs200063017	0.003	EAS:0.003	.	NEA:0.001	0.003	22.7	FA/SI	NoEarPhenotype	PMC2597962: heterozygous missense mutation in patient with Joubert syndrome including cystic kidney disease and hearing loss	No; gnomAD MAF>0.001
					Heterozygous	BCL2L10:NM_020396:exon1:c.C414A:p.C138X	rs79743288	0.0006	EAS:0.0006	.	.	.	36	MT	NoKOMouse	PMC6817556: GWAS locus for age-related hearing loss	No
					**Homozygous**	**CLDN9:NM_020982:exon1:c.C75G:p.C25W**	**rs368045321**	**0.0004**	**OTH:0.0005**	**.**	**.**	**0.004**	**20.6**	**FA/LRT/MA/mLR/mSVM/MT/PP2/PR/SI**	**PMC2720454: AR deafness with loss of sensory cells**	**PMC6745279: AR nonsyndromic hearing loss**	**No**
					**Heterozygous**	**ANKRD11:NM_013275:exon9:c.A3830G:p.E1277G**	**.**	**.**	**.**	**.**	**.**	**.**	**24.5**	**LRT/MA/MT/PP2/PR/SI**	**Het-KO mice with abnormal ABR**	**MIM148050: AD KBG syndrome, may include hearing loss in 1/3, also craniofacial dysmorphisms, susceptibility to nasopharyngeal carcinoma**	**No**
					**Heterozygous**	**FLNA:NM_001110556:exon39:c.A6350G:p.N2117S**	**rs375205247**	**0.002**	**EAS:0.002**	**.**	**.**	**.**	**20.2**	**FA/LRT/MT/PR**	**NoKOMouse**	**MIM311300: otopalatodigital syndrome inc. hearing loss**	**No; gnomAD MAF>0.001**
22	4.4	F	.	.	Potential Compound Heterozygous or Haplotype	PATJ:NM_176877:[exon11:c.C1351G:p.R451G]; [exon34:c.4461+1G>A]	rs117405402; rs199981703	0.003; 0.003	EAS:0.003; EAS:0.003	EAS:0.003; EAS:0.004	.	0.007; 0.01	25.3; 32	LRT/MA/MT/PP2/PR/SI; MT	Hom-KO mice with abnormal startle reflex (late adult)	.	Yes; gnomAD MAF>0.001
					Heterozygous	ARID1B:NM_001346813:exon20:c.G5648A:p.R1883H	rs758748419	0	FIN:0.00009	.	.	0 (SouthAsia=0.0007)	23.2	LRT/MA/MT/PR/SI	Preweaning lethality	MIM614556: AD Coffin-Siris syndrome	No
					Heterozygous	MYO3A:NM_017433:exon32:c.C4483T:p.R1495X	rs371876274	0.0002	SAS:0.001	SAS:0.002	.	.	41	LRT/MT	Hom-KO mice with abnormal ABR (het not tested)	MIM606808: DFNB30	No; gnomAD MAF>0.001; previously ruled out due to lack of second allele explaining AR hearing loss
					**Heterozygous**	**CBLN3:NM_001039771:exon3:c.C550T:p.R184C**	**rs562291434**	**0.0002**	**EAS:0.0002**	**.**	**.**	**.**	**32**	**LRT/MT/PP2/PR/SI**	**Het-KO mice with abnormal ABR**	**.**	**No**

					Heterozygous	HOXB6:NM_018952:exon3:c.T405A:p.N135K	rs758723592	.	.	.	.	.	25.6	FA/LRT/MA/mLR/mSVM/MT/PP2/PR/SI	PMID1358998: gain-of-function mutant with early postnatal lethality, craniofacial/axial skeletal anomalies, cleft palate, microtia, skull bone defects etc	.	Yes
					**Heterozygous**	**GREB1L:NM_001142966:exon22:c.C3798G:p.S1266R**	**rs954005555**	**0.0006**	**EAS:0.0006**	**.**	**.**	**0.003**	**16.6**	**LRT/MA/MT/PR/SI**	**Preweaning lethality**	**PMC7349314: AD profound sensorineural hearing loss, variable genitourinary findings**	**No**
					Heterozygous	TRMT1:NM_017722:exon4:c.C619T:p.Q207X	.	.	.	.	.	.	36	MT	Preweaning lethality; PMID33499731: subcellular relocalization of RNA modification enzymes may play a role in neuronal plasticity and transmission of information	PMID30289604: intellectual disability, microcephaly, brain anomalies; PMC7981843: includes hearing loss	No
					Heterozygous	SALL4:NM_020436:exon4:c.C2782T:p.R928C	rs201682235	0	ASJ:0.0003	AMR:0.001	.	.	27.7	LRT/MA/MT/PP2/PR/SI	PMID16790473: SALL4 haploinsufficiency led to anorectal/heart anomalies, exencephaly and kidney agenesis	MIM607343: AD sensorineural hearing loss due to Duane-radial ray syndrome or IVIC syndrome	No; 1KG MAF>0.001
23	4.61	F	.	Hearing loss, uncle of grandfather	**Potential Compound Heterozygous or Haplotype**	**CDH23:NM_022124:[exon7:c.C437T:p.P146L]; [exon27:c.G3262A:p.V1088M]; [exon48:c.G6911A:p.R2304Q]**	**rs765103490; rs200632520; rs201434373**	**NA; 0.002; 0.0015**	**NA; EAS:0.002; EAS:0.0015**	**NA; NA; EAS:0.001**	**.**	**0.001; 0.003; 0.007**	**24.7; 24.3; 22.7**	**LRT/MT/PP2/PR/SI; LRT/MA/mLR/mSVM/MT/PP2/SI; MT/SI**	**KO-hom abnormal righting response, abnormal gait, absent pinna reflex; PMID11138008: disrupted stereocilia organization**	**MIM605516: DFNB12; AR Usher 1D**	**No; gnomAD MAF>0.001; previously ruled out due to lack of second allele explaining AR hearing loss**
					Potential Compound Heterozygous or Haplotype	ERCC4:NM_005236:[exon8:c.A1483G:p.T495A]; [exon11:c.C2545G:p.Q849E]	NA; rs374186605	NA; 0.001	NA; EAS:0.001	.	.	NA; 0.01	21.9; 22	LRT/MA/MT; LRT/MA/MT/PP2	Preweaning lethality	MIM278760: AR Cockayne syndrome with hearing loss	No; gnomAD MAF>0.001
					**Heterozygous**	**MYO18B:NM_032608:exon8:c.G1982A:p.W661X**	**rs372939044**	**0**	**AFR:0.0005**	**.**	**.**	**.**	**44**	**LRT/MT**	**Het-KO mice with abnormal ABR**	**MIM616549: AR Klippel-Feil syndrome with nemaline myopathy and facial dysmorphism; PMC5885878: hearing loss common in KFS; PMID18722888: 60% of Klippel-Feil HL patients with ear anomalies including inner ear dysplasia and deformed internal acoustic canal**	**No**
24	6.1	M	EVA, B; fever, jaundice, foul umbilical discharge and apneic episodes with antibiotics and phototherapy in neonatal period	.	**Hemizygous**	**FLNA:NM_001110556:exon39:c.A6350G:p.N2117S**	**rs375205247**	**0.002**	**EAS:0.002**	**.**	**.**	**.**	**20.2**	**FA/LRT/MT/PR**	**NoKOMouse**	**MIM311300: otopalatodigital syndrome inc. hearing loss; PMC2957847; PMID15654694: Melnick-Needles syndrome includes skull base sclerosis**	**No; gnomAD MAF>0.001**
					Hemizygous	*G6PD:NM_000402:exon11:c.C1450T:p.R484C*	*rs398123546*	*0.0006*	*SAS:0.0007*	*SAS:0.001*	*.*	*.*	*31*	*FA/LRT/MA/mLR/mSVM/MT/PP2/PR/SI*	*PMC7744953: G6pd overexpression protects from hearing loss progression*	*MIM300908: hemolytic anemia*	*No; 1KG MAF>0.001*
27	7.72	F	EVA, B; OM, L	Hearing loss, 2nd-degree cousin, paternal side	*Heterozygous*	*MUC1:NM_001204288:exon6:c.G453A:p.W151X*	*rs369649894*	*0*	*LAT:0.00007*	*.*	*.*	*0.009*	*8.35*	*PP2/PR/SI*	*NoKOMouse*	*MIM158340: AD kidney disease; PMID12560150: Muc1 expressed in MEE from patients with otitis media*	*No*
					Heterozygous	ADAM28:NM_014265:exon7:c.577-1G>A	rs370669549	0.0002	EAS:0.0002	.	.	0.006	32	MT	NoKOMouse	PMID23640157: ADAM28 was screened but was negative for AD progressive bone disorder of skull which may include nerve entrapment leading to hearing loss	No
					Potential Compound Heterozygous or Haplotype	MADD:[NM_130470:exon14:c.T2458G:p.S820A]; [exon26:c.T4046A:p.V1349E]	rs778218513; rs756795627	0.0002; 0.0002	EAS:0.0002; EAS:0.0002	.	.	NA; 0.006	27.2; 23.4	LRT/MT/PP2/SI; MT/PP2/PR/SI	NoKOMouse	MIM603584: AR neurodevelopmental disorder with dysmorphic facies, impaired speech, hypotonia	No
					**Potential Compound Heterozygous or Haplotype**	**GDPD5:NM_030792:[exon8:c.G554A:p.R185H]; [exon7:c.C404T:p.T135M]**	**rs745585758; rs373413383**	**0.002; 0**	**ME:0.003; AFR:0.00002**	**.**	**.**	**0 (SouthAsia=0.0007); NA**	**23.1; 24.8**	**LRT/MT/PP2; LRT/MA/MT/PP2**	**Hom-KO mice with abnormal ABR (het not tested)**	**.**	**No; gnomAD MAF>0.001**
					Heterozygous	*SNAP29:NM_004782:exon5:c.C766T:p.R256X*	*rs148156702*	*0.001*	*EAS:0.001*	*EAS:0.001*	*.*	*.*	*38*	*LRT/MT*	*Het-KO mice with increased BMD*	*MIM609528: AR cerebral dysgenesis, neuropathy, ichthyosis, palmoplantar keratoderma syndrome*	*No; gnomAD MAF>0.001*
